# Analysis of astigmatism type influence on *in vivo* corneal biomechanical measurements

**DOI:** 10.3389/fbioe.2026.1903049

**Published:** 2026-09-01

**Authors:** Yan Huo, Qiyu Zhang, Zhengyuan Qu, Ruisi Xie, Yutong Li, Haohan Zou, Guoxing Zhao, Yan Wang

**Affiliations:** 1 School of Medicine, Nankai University, Tianjin, China; 2 Clinical College of Ophthalmology, Tianjin Medical University, Tianjin, China; 3 Tianjin Eye Hospital, Tianjin Key Lab of Ophthalmology and Visual Science, Tianjin Eye Institute, Nankai University Affiliated Eye Hospital, Tianjin, China; 4 Nankai University Eye Institute, Nankai University, Tianjin, China

**Keywords:** astigmatism, astigmatism type, corneal biomechanics, *in vivo* measurement, precision evaluation

## Abstract

**Purpose:**

This study aimed to evaluate the influence of astigmatism type on *in vivo* corneal biomechanical measurements and to analyze the correlation using astigmatic power vector components, providing evidence for precise clinical interpretation.

**Methods:**

This prospective observational comparative study included 155 eyes from 155 participants. Corneal tomographic and biomechanical parameters were obtained using Pentacam and Corvis ST. Participants were classified into four groups according to astigmatism axis and cylinder power: no astigmatism (n = 30), with-the-rule astigmatism (WTR, n = 50), against-the-rule astigmatism (ATR, n = 37), and oblique astigmatism (Oblique, n = 38). Cylinder power and axis were decomposed into Thibos power vector components (J0, J45). Differences in 15 dynamic corneal response (DCR) parameters among the four groups were compared using one-way analysis of covariance (ANCOVA). Partial correlation analysis and multiple linear regression were performed to evaluate the correlations between J0/J45 and DCR parameters after adjusting for confounding factors, followed by within-subgroup analyses.

**Results:**

After adjustment for intraocular pressure, central corneal thickness, and age, the stiffness parameter SP-A1 differed significantly among the four groups (P = 0.007). The WTR group had higher SP-A1 than the ATR group (mean diff = 4.89 mmHg/mm, P = 0.0284) and the Oblique group (mean diff = 5.32 mmHg/mm, P = 0.0122). Multiple linear regression showed that J0 was independently correlated with Integrated Radius (β = 0.196), HC dArc length (β = 0.223), SP-A1 (β = 0.101), Radius (β = −0.182) and SSI (β = −0.181, all P < 0.05). J45 was independently associated with A1 time (β = −0.033) and A2 velocity (β = −0.156, both P < 0.05).

**Conclusion:**

*In vivo* biomechanical measurements were influenced by different astigmatism types. SP-A1 was the primary outcome and differed significantly among astigmatism types. Power vector analysis showed that a greater WTR component was correlated with higher SP-A1, Integrated Radius, and HC dArc length, and lower Radius. Astigmatism axis type and cylinder power could be considered when interpreting *in vivo* biomechanical measurements to improve the precision of corneal biomechanical evaluation.

## 1 Introduction

Corneal biomechanical properties are essential for maintaining ocular shape, supporting optical imaging, and preserving visual function (Thomasy et al., 2024). Previous studies have shown that changes in corneal biomechanical properties are closely related to the occurrence and progression of corneal ectatic diseases, the safety and accuracy of refractive surgery, and the evaluation of corneal cross-linking efficacy (Roberts and Dupps, 2014; Bao et al., 2016; Sedaghat et al., 2018; Chen et al., 2023; Huo et al., 2024b). Therefore, accurate *in vivo* measurement and evaluation of corneal biomechanics are important. Precise interpretation of clinical biomechanical measurements is critical for improving the reliability of corneal disease risk identification and enhancing the management of refractive surgery (Jacobs et al., 2023; Wang et al., 2023; 2025; Zhang et al., 2025).

Corneal Visualization Scheimpflug Technology (Corvis ST, Oculus, Wetzlar, Germany) is a widely used clinical device for *in vivo* corneal biomechanical measurement (De Stefano and Dupps, 2017; Yu et al., 2020); it uses an air puff to induce corneal deformation and generates dynamic corneal response (DCR) parameters that indirectly characterize corneal biomechanical properties (Baptista et al., 2021). Notably, the cornea is not perfectly spherical. Curvature varies across meridians, giving rise to astigmatism (Gurnani and Kaur, 2026). Astigmatism magnitude and meridional curvature may affect the precise characterization of *in vivo* corneal biomechanical properties (Nemeth et al., 2017; Liu et al., 2022). Li et al. conducted a preliminary analysis of the relationship between astigmatism type and *in vivo* biomechanical parameters in cataract patients, suggesting that astigmatism type may influence DCR measurements (Li et al., 2025). However, the current study was limited to a cataract population; no refined vector analysis was conducted, and it did not adjust for confounders such as age, central corneal thickness (CCT), and intraocular pressure (IOP) (Vinciguerra et al., 2016; Guo et al., 2024). Therefore, it is important to refine astigmatism type classification and to evaluate its magnitude relative to *in vivo* measurements after adjusting for confounding factors, for more precise characterization of *in vivo* biomechanics.

This study systematically evaluated whether different astigmatism types influence DCR parameters measurements after adjusting for confounding factors. Furthermore, the power vector method was used to analyze the associations of astigmatic axis and magnitude with biomechanical parameters, thereby facilitating more precise clinical interpretation of *in vivo* corneal biomechanical parameters.

## 2 Methods

### 2.1 Participant inclusion and exclusion criteria

This prospective observational comparative study included 155 patients who underwent preoperative evaluation for refractive surgery at Tianjin Eye Hospital, from January 2023 to February 2026. The study was approved by the Tianjin Eye Hospital Ethics Committee (KY-2023068), followed the principles of the Declaration of Helsinki, and obtained signed informed consent for data use from all patients.

All participants underwent comprehensive examinations, including uncorrected visual acuity, best-corrected visual acuity, objective refraction, manifest refraction, noncontact intraocular pressure test, axial length measurement, slit-lamp, corneal tomography (Pentacam, Oculus, Wetzlar, Germany), and Corvis ST examination. Inclusion criteria: 1) Completion of Pentacam and Corvis ST examinations with acceptable quality; 2) Complete data for age, sex, IOP, and major clinical parameters; 3) No anterior segment abnormalities on slit-lamp biomicroscopy that may affect corneal morphology or imaging quality; 4) No morphological evidence of keratoconus or obvious ectasia risk; 5) No history of ocular surgery or ocular trauma; 6) Stop wearing soft corneal contact lenses within 2 weeks or rigid contact lenses (including orthokeratology lenses) within 4 weeks before the evaluation. All criteria were independently assessed by two experienced specialists (YW and YH).

### 2.2 Astigmatism type and vector decomposition

**FIGURE 1:**
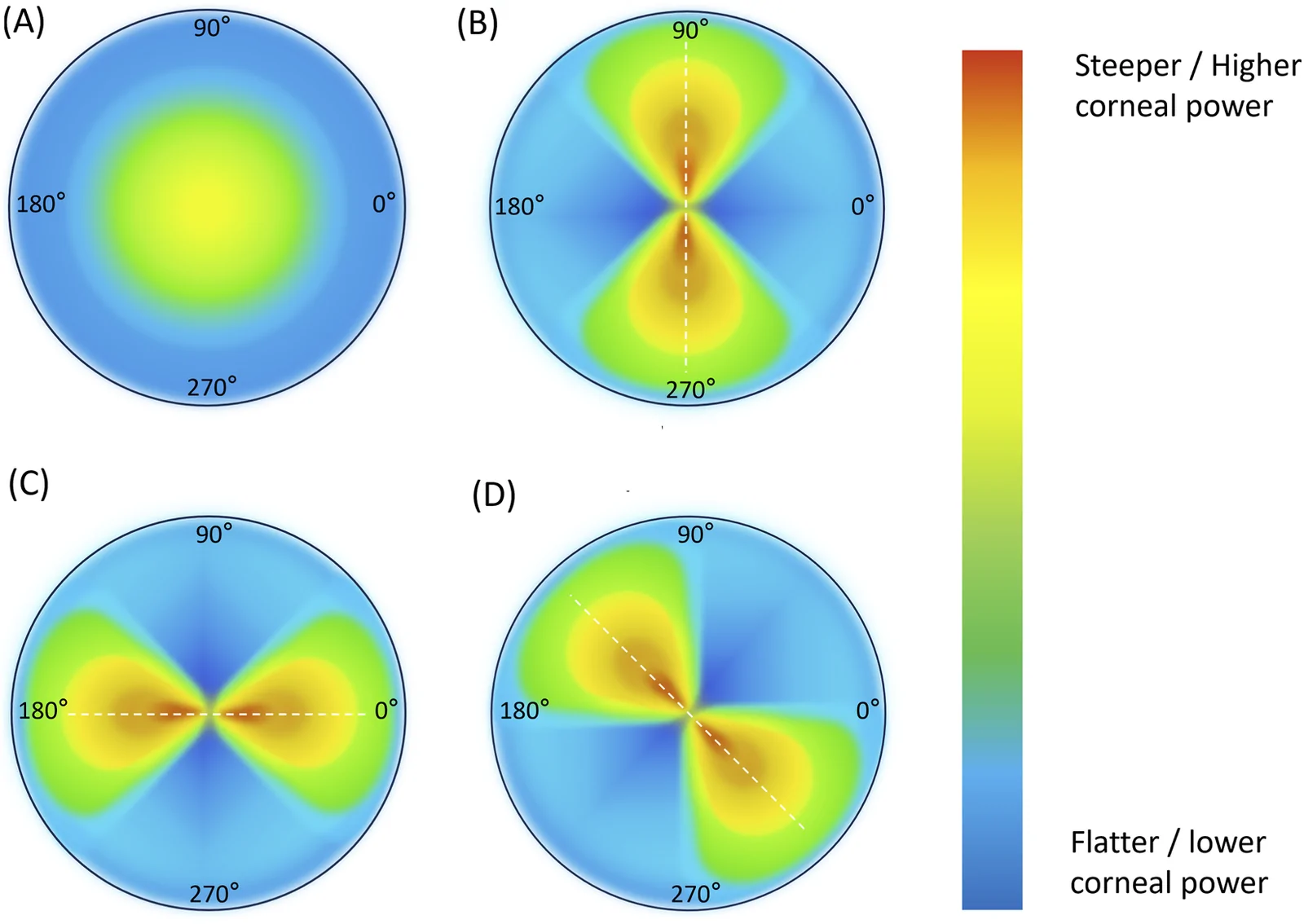
Schematic axial power maps of the four astigmatism groups. WTR = with-the-rule astigmatism; ATR = against-the-rule astigmatism; Oblique = oblique astigmatism; D = diopter. **(A)** No astigmatism: near round. **(B)** WTR: 60° ≤ steep meridian axis ≤ 120°. **(C)** ATR: steep meridian axis ≤ 30° or steep meridian axis ≥ 150°. **(D)** Oblique: 30° < steep meridian axis < 60° or 120° < steep meridian axis < 150°.

Astigmatism types were defined according to the axis of the steepest meridian. Based on the ‘Astig’ and ‘axis’ values in the original CSV file exported from Pentacam, eyes were classified into four types: 1. No astigmatism was defined as cylinder power <0.25 diopter (D); When the cylinder power was ≥0.25 D, 2. With-the-rule astigmatism (WTR) was defined as the steepest meridian located between 60° and 120°; 3. Against-the-rule astigmatism (ATR) was defined as the steepest meridian located between 0° and 30° or between 150° and 180°; 4. Oblique astigmatism was defined as the steepest meridian located between 30° and 60° or between 120° and 150° (Ueno et al., 2021) (Figure 1).

To retain information on both astigmatism orientation and cylinder power, Thibos power vector analysis was further used for vector decomposition. We used the absolute value of negative cylinder power in the analysis, and J0/J45 were calculated as follows (Thibos and Horner, 2001):

$$J_{0}=\left(\left|C\right|/2\right)\times \cos\left(2\theta \right)$$

$$J_{45}=\left(\left|C\right|/2\right)\times \sin\left(2\theta \right)$$

In these formulas, C denotes the cylinder power, and θ denotes the negative cylinder axis in degrees. J0 represents the horizontal/vertical vector component, with J0 > 0 indicating a WTR bias and J0 < 0 indicating an ATR bias; J45 represents the oblique component of astigmatism.

### 2.3 Corvis ST measurements

Corvis ST parameters were exported automatically by the device. Based on previous studies (Huo et al., 2024a; Huo et al., 2024c), 15 DCR parameters were included: A1 time, A2 time, HC time, A1 velocity, A2 velocity, A1 dArc length, A2 dArc length, HC dArc length, DA Ratio Max 1 mm, DA Ratio Max 2 mm, Radius, Integrated Radius, Max inverse radius, SP-A1, and SSI. The analyzed data were obtained from the original CSV files. Only measurements with an “OK” quality status were included. If multiple scans were performed on the same day, the mean value was used. Parameter definitions are provided in Supplementary Table S1.

### 2.4 Statistical analysis

All statistical analyses were performed using IBM SPSS Statistics 27.0 (IBM Corp., Armonk, NY, United States) and Python 3.11.0. Continuous variables are presented as mean ± SD, whereas categorical variables as counts. The Shapiro-Wilk test was used to check the normality of continuous variables. For between-group comparisons and correlation analyses, normally distributed variables were analyzed using one-way analysis of variance (ANOVA) and Pearson correlation analysis, non-normally distributed variables were analyzed using the Kruskal-Wallis test and Spearman correlation analysis, and categorical variables were analyzed using Fisher’s exact test. One-way analysis of covariance (ANCOVA) was used to evaluate differences in biomechanical parameters among the four groups after adjusting for age, CCT, and IOP. Partial correlation analysis and multivariable linear regression were applied as exploratory analyses to examine the relationships of J0 and J45 with biomechanical parameters, while controlling for the same confounding factors. Regression diagnostics included the Shapiro–Wilk test for residual normality, variance inflation factors (VIFs) for multicollinearity, the Breusch–Pagan test for homoscedasticity, and Cook’s distance for influential observations. HC3 robust standard errors were used when residuals deviated from normality. Based on the between-group distribution of SP-A1 reported in a previous study (Li et al., 2025), the estimated effect size was approximately Cohen’s f = 0.45. Considering differences in the study populations, a more conservative effect size of f = 0.40 was adopted. With four groups, a two-sided α of 0.05, and 90% statistical power, at least 24 eyes per group were required. After allowing for a 15% rate of unusable or incomplete data, the target sample size was 29 eyes per group. The final study included 155 eyes, with at least 30 eyes in each group. P < 0.05 was considered statistically significant. Post hoc comparisons for subgroups were adjusted using the Holm-Bonferroni method, and other exploratory analyses were performed using Fisher’s least significant difference (LSD) method.

## 3 Results

### 3.1 Baseline characteristics

**FIGURE 2:**
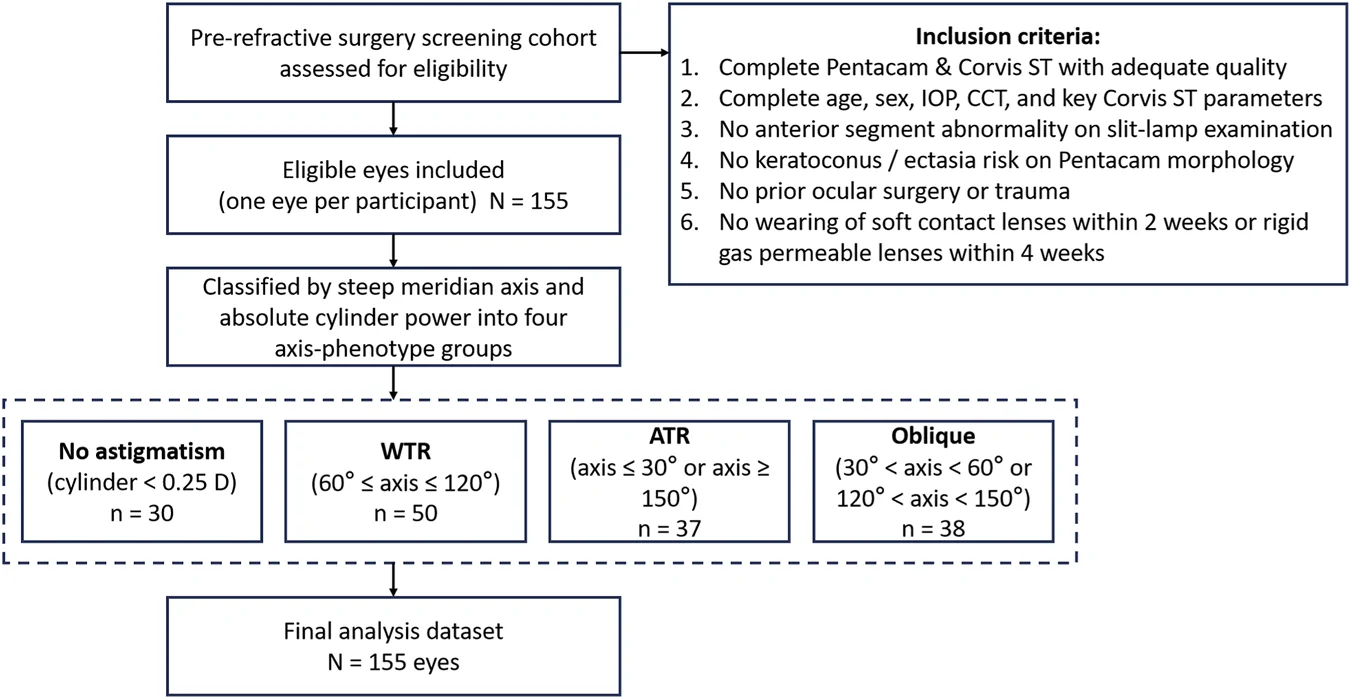
Sample selection flow and analytic cohort. WTR = with-the-rule astigmatism; ATR = against-the-rule astigmatism; Oblique = oblique astigmatism; D = diopter; CCT = central corneal thickness; IOP = intraocular pressure.

The study workflow is shown in Figure 2. A total of 155 eyes from 155 patients with myopia were included. The four groups comprised 30 eyes with no astigmatism, 50 eyes with WTR, 37 eyes with ATR, and 38 eyes with Oblique.

**FIGURE 3:**
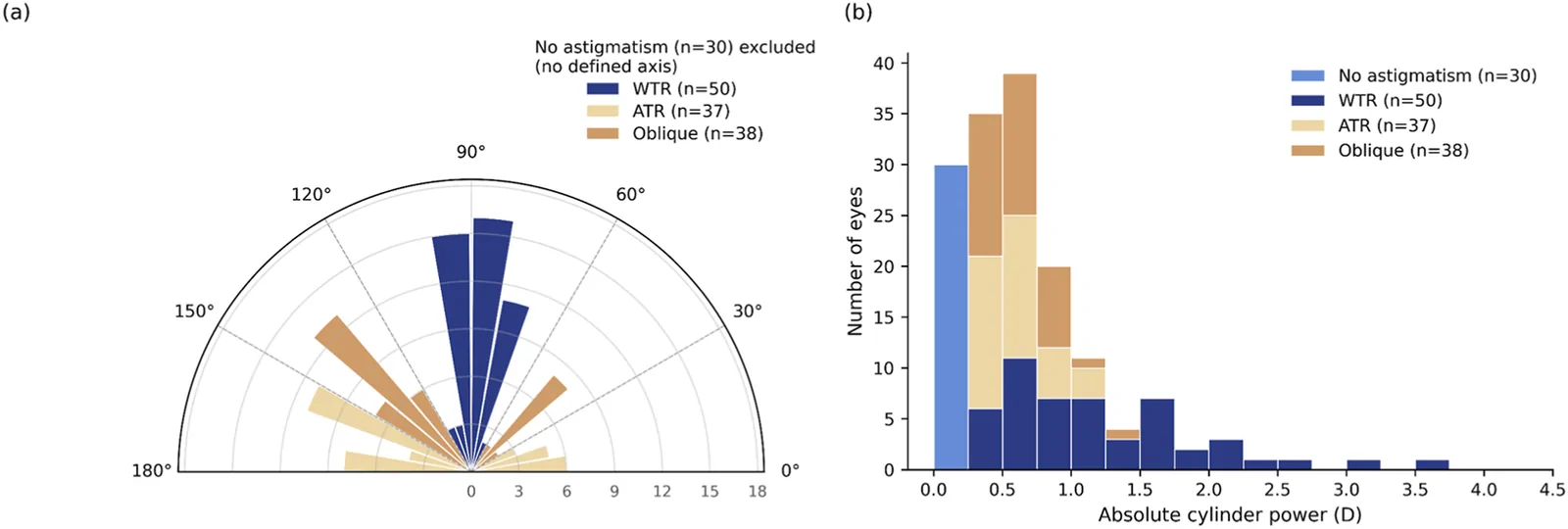
Distribution of steep meridian axis and absolute cylinder power. WTR = with-the-rule astigmatism; ATR = against-the-rule astigmatism; Oblique = oblique astigmatism; D = diopter. **(a)** Steep meridian axis distribution (n eyes per 10° bin). **(b)** Absolute cylinder power distribution.

Baseline demographic data showed significant differences in cylinder power and age among the four groups (P < 0.001), whereas no significant differences were observed in the other variables (Table 1). The distributions of the steep meridian axis and absolute cylinder power are shown in Figure 3.

**TABLE 1:** Baseline characteristics.

Variable	No astigmatism (n = 30)	WTR (n = 50)	ATR (n = 37)	Oblique (n = 38)	p value
Age (years)	26.32 ± 7.66	21.38 ± 4.48	26.98 ± 8.32	24.81 ± 5.77	<0.001^a^
Sphere (D)	−4.59 ± 1.53	−4.54 ± 1.65	−4.26 ± 1.64	−4.97 ± 1.46	0.241^a^
Cylinder (D)	0.15 ± 0.06	1.09 ± 0.75	0.47 ± 0.23	0.49 ± 0.24	<0.001^a^
SER (D)	−4.67 ± 1.33	−5.08 ± 1.58	−4.50 ± 1.64	−5.21 ± 1.48	0.102^a^
IOP (mmHg)	17.13 ± 2.48	17.38 ± 2.28	17.78 ± 2.90	18.33 ± 2.30	0.190^a^
CCT (μm)	554.67 ± 18.37	557.30 ± 29.94	560.43 ± 26.63	565.08 ± 25.45	0.302^a^
Sex (M/F)	15/15	35/15	18/19	18/20	0.093^b^

^a^P stands for the Kruskal-Wallis test, and.

^b^P stands for the Fisher’s test. WTR, with-the-rule astigmatism; ATR, against-the-rule astigmatism; Oblique = oblique astigmatism; D = diopter; CCT, central corneal thickness; IOP, intraocular pressure; SER, spherical equivalent refraction.

### 3.2 Astigmatism type and dynamic corneal response parameters analysis

Correlation analyses examined the relation of age, CCT, IOP, SER, K1, K2, axial length, sex, and myopia severity with the 15 DCR parameters (Supplementary Table S2). Finally, Age, CCT, and IOP were therefore included as covariates.

After adjustment for age, IOP, and CCT, SP-A1 showed a significant difference among the four groups (P = 0.0071) in ANCOVA (Table 2; Supplementary Table S3), whereas the other 14 parameters did not differ significantly (all P > 0.05). After Holm-Bonferroni adjustment, the WTR group had significantly higher SP-A1 than the ATR group (mean diff = 4.89 mmHg/mm, 95% CI, 1.46 to 8.33; P = 0.0284) and the Oblique group (mean diff = 5.32 mmHg/mm, 95% CI, 1.95 to 8.70; P = 0.0122). No significant differences in biomechanical parameters were observed between no astigmatism group and other groups, or between the ATR and Oblique groups (all P > 0.05).

**TABLE 2:** Covariate-adjusted comparisons of dynamic corneal response parameters among the four astigmatism types.

Parameter	No astigmatism (n = 30)	WTR (n = 50)	ATR (n = 37)	Oblique (n = 38)	F	p value
A1 time (ms)	7.556 ± 0.008 (7.495 ± 0.283)	7.572 ± 0.006 (7.539 ± 0.261)	7.554 ± 0.007 (7.568 ± 0.344)	7.553 ± 0.007 (7.630 ± 0.277)	2.084	0.1048
A2 time (ms)	22.004 ± 0.046 (22.073 ± 0.331)	21.945 ± 0.037 (21.977 ± 0.381)	21.907 ± 0.042 (21.894 ± 0.551)	22.011 ± 0.041 (21.926 ± 0.365)	1.453	0.2297
HC time (ms)	17.246 ± 0.079 (17.260 ± 0.379)	17.207 ± 0.062 (17.193 ± 0.480)	17.156 ± 0.071 (17.168 ± 0.401)	17.214 ± 0.070 (17.210 ± 0.394)	0.26	0.8544
A1 velocity (m/s)	0.144 ± 0.002 (0.148 ± 0.024)	0.144 ± 0.001 (0.145 ± 0.018)	0.147 ± 0.002 (0.147 ± 0.023)	0.146 ± 0.001 (0.141 ± 0.018)	1.43	0.2364
A2 velocity (m/s)	−0.258 ± 0.005 (−0.262 ± 0.034)	−0.253 ± 0.004 (−0.255 ± 0.030)	−0.256 ± 0.004 (−0.255 ± 0.034)	−0.254 ± 0.004 (−0.249 ± 0.030)	0.315	0.8144
A1 dArc length (mm)	−0.020 ± 0.000 (−0.020 ± 0.002)	−0.020 ± 0.000 (−0.019 ± 0.002)	−0.020 ± 0.000 (−0.021 ± 0.002)	−0.020 ± 0.000 (−0.021 ± 0.003)	1.06	0.3681
A2 dArc length (mm)	−0.025 ± 0.001 (−0.025 ± 0.006)	−0.026 ± 0.001 (−0.025 ± 0.004)	−0.025 ± 0.001 (−0.026 ± 0.007)	−0.025 ± 0.001 (−0.025 ± 0.004)	0.28	0.8395
HC dArc length (mm)	−0.140 ± 0.003 (−0.141 ± 0.020)	−0.138 ± 0.003 (−0.138 ± 0.023)	−0.144 ± 0.003 (−0.144 ± 0.022)	−0.142 ± 0.003 (−0.140 ± 0.018)	0.71	0.5474
DA ratio max (1 mm)	1.545 ± 0.006 (1.551 ± 0.033)	1.543 ± 0.004 (1.548 ± 0.044)	1.551 ± 0.005 (1.547 ± 0.040)	1.550 ± 0.005 (1.542 ± 0.043)	0.649	0.5847
DA ratio max (2 mm)	4.121 ± 0.041 (4.178 ± 0.289)	4.090 ± 0.033 (4.120 ± 0.347)	4.195 ± 0.037 (4.181 ± 0.320)	4.125 ± 0.037 (4.054 ± 0.313)	1.487	0.2204
Radius (mm)	7.729 ± 0.134 (7.666 ± 0.657)	7.839 ± 0.107 (7.750 ± 0.748)	7.956 ± 0.121 (8.010 ± 0.904)	7.826 ± 0.119 (7.938 ± 0.900)	0.548	0.6502
Integrated radius (mm^1^)	7.591 ± 0.113 (7.744 ± 0.708)	7.685 ± 0.089 (7.775 ± 0.987)	7.473 ± 0.102 (7.430 ± 0.967)	7.571 ± 0.100 (7.374 ± 0.791)	0.791	0.5006
Max inverse radius (mm^1^)	0.161 ± 0.003 (0.162 ± 0.014)	0.164 ± 0.003 (0.165 ± 0.019)	0.164 ± 0.003 (0.163 ± 0.024)	0.160 ± 0.003 (0.158 ± 0.015)	0.628	0.5978
SP-A1 (mmHg/mm)	114.909 ± 1.414 (112.188 ± 13.224)	117.195 ± 1.122 (114.831 ± 15.477)	112.305 ± 1.277 (113.623 ± 16.349)	111.873 ± 1.253 (115.849 ± 14.604)	4.18	0.0071* 0.0284*^a^ 0.0122*^b^ >0.05^c^
SSI	0.881 ± 0.023 (0.881 ± 0.132)	0.866 ± 0.018 (0.853 ± 0.128)	0.896 ± 0.021 (0.906 ± 0.138)	0.907 ± 0.020 (0.916 ± 0.119)	0.834	0.4773

Values outside parentheses are covariate-adjusted estimated marginal means ± standard errors after adjustment for age, central corneal thickness (CCT), and intraocular pressure (IOP); values in parentheses are unadjusted raw descriptive means ± standard deviations. * indicates P < 0.05. WTR, with-the-rule astigmatism; ATR, against-the-rule astigmatism; Oblique = oblique astigmatism; D = diopter.

^a^ P indicates the Holm-Bonferroni adjusted comparison between the WTR, and ATR, groups.

^b^ P indicates the Holm-Bonferroni adjusted comparison between WTR, and Oblique.

^c^ P indicates that no statistically significant differences remained among the other four pairwise comparisons after Holm-Bonferroni adjustment.

### 3.3 Vector analysis

After adjustment for age, CCT, and IOP, partial correlation analysis showed that J0 was positively correlated with SP-A1 (rho = 0.220, P = 0.006), A1 time (rho = 0.158, P = 0.049), and A1 dArc length (rho = 0.179, P = 0.026), and negatively correlated with A1 velocity (rho = −0.183, P = 0.022); J45 was negatively correlated only with SP-A1 (rho = −0.164, P = 0.042), and was not significantly correlated with the other 14 parameters (all P > 0.05) (Table 3).

**TABLE 3:** Primary vector correlations with Corvis ST parameters.

Parameter	Rho (J0)	p value (J0)	Rho (J45)	p value (J45)
A1 time (ms)	+0.158	0.0493*	−0.028	0.7338
A2 time (ms)	−0.065	0.4202	−0.026	0.7525
HC time (ms)	+0.047	0.5653	−0.002	0.9851
A1 velocity (m/s)	−0.183	0.0225*	−0.051	0.5290
A2 velocity (m/s)	−0.003	0.9739	−0.091	0.2599
A1 dArc length (mm)	+0.179	0.0255*	−0.130	0.1060
A2 dArc length (mm)	+0.009	0.9110	+0.115	0.1555
HC dArc length (mm)	+0.064	0.4268	−0.020	0.8101
DA ratio max (1 mm)	−0.030	0.7072	−0.017	0.8383
DA ratio max (2 mm)	−0.134	0.0957	−0.024	0.7670
Radius (mm)	−0.021	0.8006	−0.039	0.6343
Integrated radius (mm^-1^)	+0.113	0.1622	−0.034	0.6789
Max inverse radius (mm^-1^)	+0.089	0.2720	−0.080	0.3230
SP-A1 (mmHg/mm)	+0.220	0.0060*	−0.164	0.0416*
SSI	−0.132	0.1025	+0.072	0.3741

* indicate P < 0.05.

**FIGURE 4:**
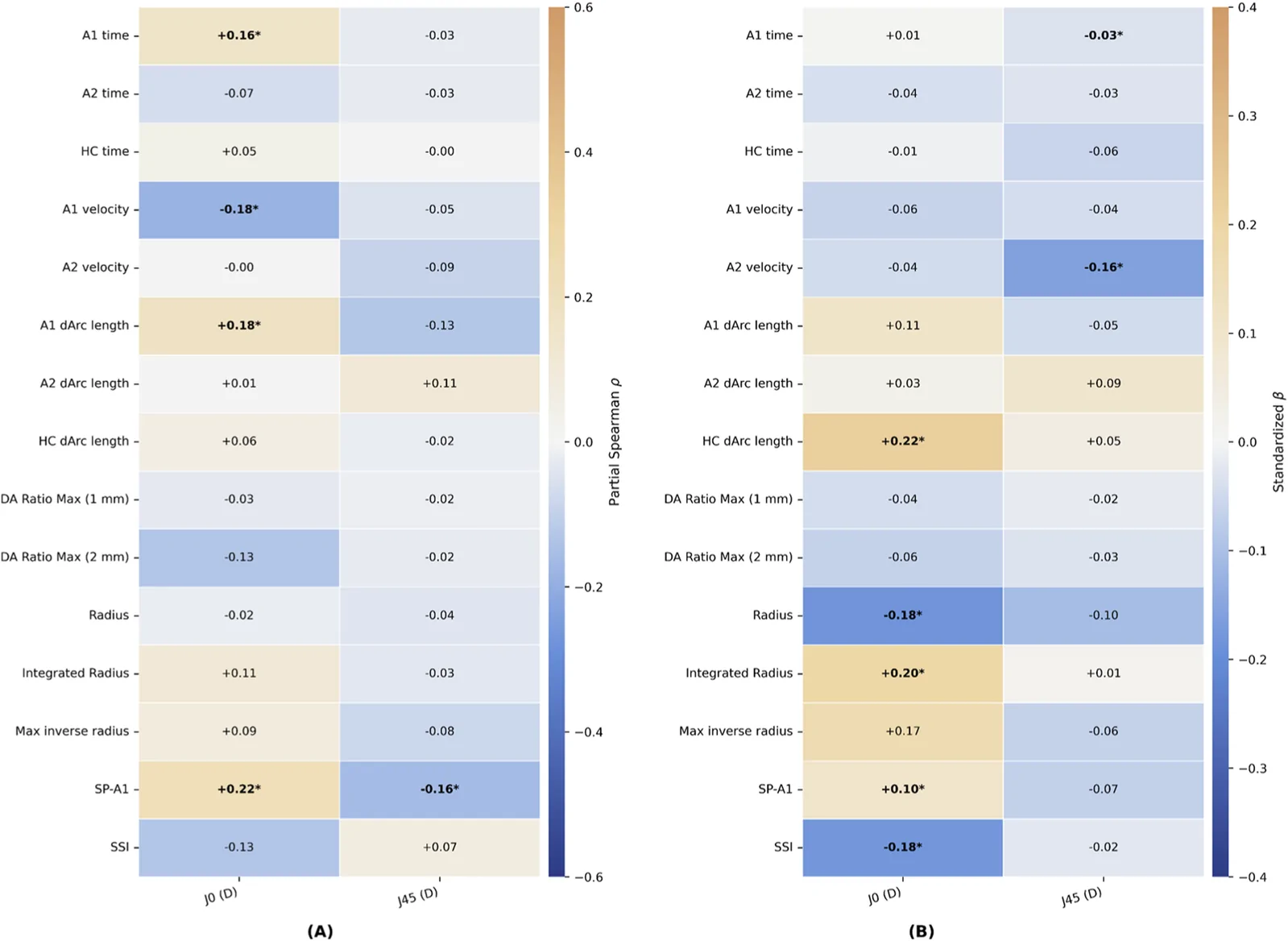
Heatmaps of Thibos J0/J45 associations with dynamic corneal response parameters. **(A)** Partial Spearman correlation coefficients (ρ). **(B)** Standardized β coefficients from multivariable linear regression. * indicates P < 0.05. CCT = central corneal thickness; IOP = intraocular pressure.

Multivariable linear regression showed that, after simultaneous inclusion of J45, age, CCT, and IOP, J0 was independently and positively associated with Integrated Radius (β = 0.196, P = 0.0012), HC dArc length (β = 0.223, P = 0.0051), and SP-A1 (β = 0.101, P = 0.0312), and independently and negatively associated with Radius (β = −0.182, P = 0.0234) and SSI (β = −0.181, P = 0.035). J45 was independently and negatively associated with A1 time (β = −0.033, P = 0.011) and A2 velocity (β = −0.156, P = 0.0242) (Figure 4; Supplementary Table S4). Model details are reported in Supplementary Table S5. The Shapiro–Wilk test indicated that residuals deviated from normality in 11 of the 15 models; no significant deviation was observed in the A1 velocity, A2 velocity, DA Ratio Max (2 mm), or SSI models (P = 0.136–0.358). VIFs for J0, J45, age, CCT, and IOP ranged from 1.128 to 1.268. Breusch–Pagan tests were not statistically significant for any model (χ^2^ = 2.887–11.024; all P ≥ 0.051). Maximum Cook’s distances ranged from 0.055 to 0.214, and no observation had a Cook’s distance >1. HC3 robust standard errors did not change the significance of the seven J0/J45 associations (p = 0.0126–0.0359).

### 3.4 Subgroup analysis

**FIGURE 5:**
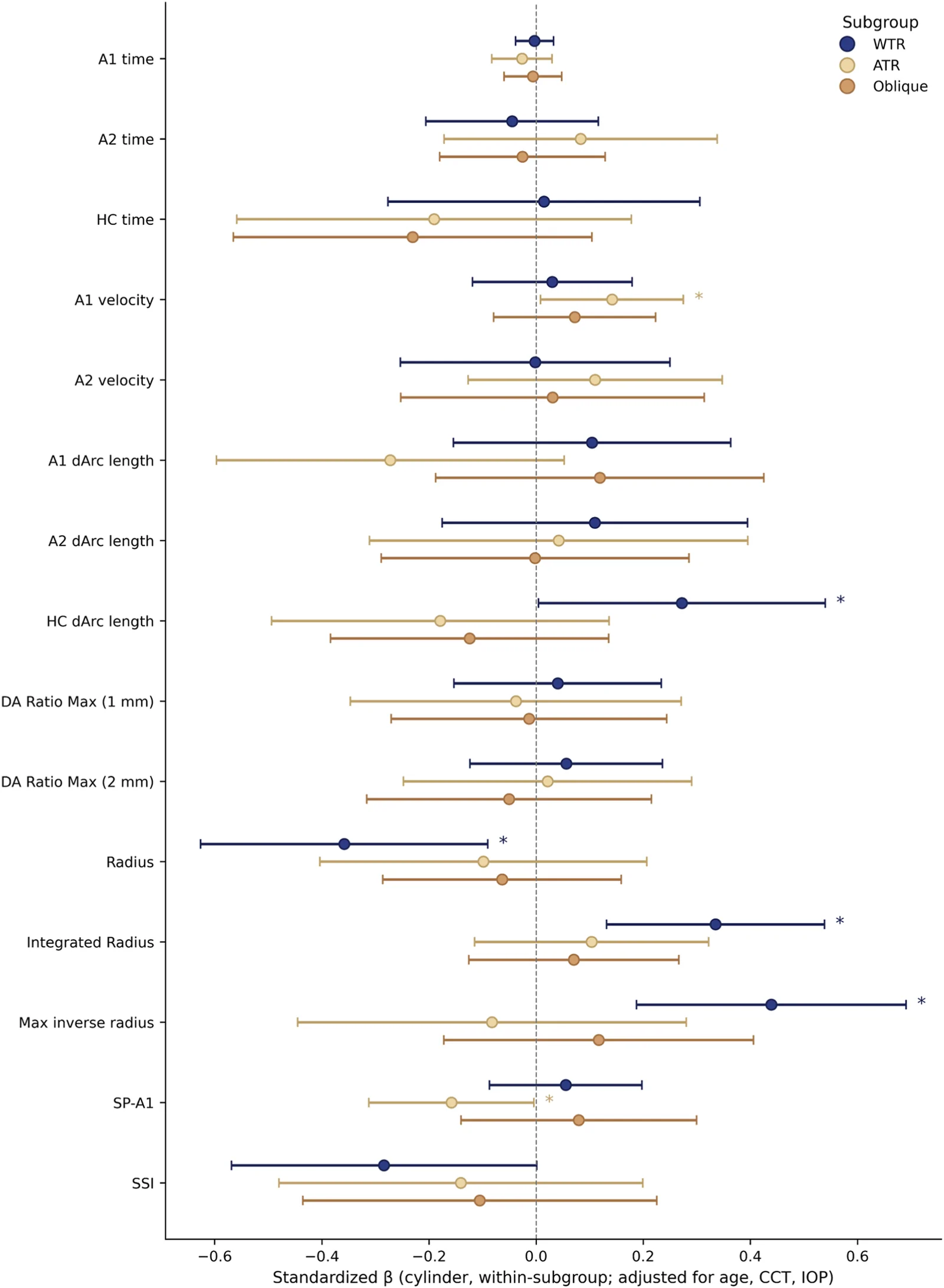
Subgroup-specific correlation between cylinder power and dynamic corneal response parameters. * indicate P < 0.05. WTR = with-the-rule astigmatism; ATR = against-the-rule astigmatism; Oblique = oblique astigmatism; CCT = central corneal thickness; IOP = intraocular pressure.

As Figure 5; Table 4 shown, within the WTR group, cylinder power was significantly and positively correlated with Integrated Radius and Max inverse radius (β = 0.335, P = 0.0018; β = 0.439, P = 0.0010), positively correlated with HC dArc length (β = 0.272, P = 0.0466), and negatively correlated with Radius (β = −0.358, P = 0.0099). Within the ATR group, cylinder power was positively correlated with A1 velocity (β = 0.142, P = 0.0379) and negatively correlated with SP-A1 (β = −0.158, P = 0.0447). Within the Oblique group, cylinder power showed no significant correlation with any Corvis ST DCR parameter (all P > 0.05).

**TABLE 4:** Subgroup associations between cylinder power and dynamic corneal response parameters within astigmatic types.

Parameter	WTR standardized β	WTR B (95% CI)	WTR p value	ATR Standardized β	ATR B (95% CI)	ATR p value	Oblique standardized β	Oblique B (95% CI)	Oblique p value
A1 time (ms)	−0.003	−0.0010 (−0.0133, +0.0113)	0.8693	−0.026	−0.0388 (−0.1213, +0.0438)	0.3462	−0.006	−0.0068 (−0.0682, +0.0546)	0.8234
A2 time (ms)	−0.045	−0.0228 (−0.1046, +0.0590)	0.5768	+0.083	+0.1958 (−0.4043, +0.7959)	0.511	−0.026	−0.0384 (−0.2707, +0.1939)	0.7387
HC time (ms)	+0.015	+0.0094 (−0.1766, +0.1955)	0.9192	−0.190	−0.3257 (−0.9558, +0.3044)	0.3003	−0.230	−0.3738 (−0.9171, +0.1695)	0.1709
A1 velocity (m/s)	+0.030	+0.0007 (−0.0029, +0.0044)	0.6847	+0.142*	+0.0139 (+0.0008, +0.0269)	0.0379	+0.072	+0.0052 (−0.0057, +0.0161)	0.3388
A2 velocity (m/s)	−0.002	−0.0001 (−0.0102, +0.0101)	0.9892	+0.110	+0.0162 (−0.0187, +0.0511)	0.3514	+0.031	+0.0038 (−0.0311, +0.0387)	0.8267
A1 dArc length (mm)	+0.104	+0.0003 (−0.0004, +0.0010)	0.421	−0.272	−0.0021 (−0.0047, +0.0004)	0.0975	+0.119	+0.0014 (−0.0021, +0.0049)	0.4356
A2 dArc length (mm)	+0.110	+0.0007 (−0.0010, +0.0024)	0.4424	+0.042	+0.0013 (−0.0098, +0.0124)	0.8097	−0.002	−0.0000 (−0.0053, +0.0052)	0.9897
HC dArc length (mm)	+0.272*	+0.0085 (+0.0001, +0.0168)	0.0466	−0.179	−0.0168 (−0.0463, +0.0127)	0.2556	−0.124	−0.0093 (−0.0287, +0.0101)	0.3373
DA ratio max (1 mm)	+0.040	+0.0024 (−0.0091, +0.0139)	0.6768	−0.038	−0.0065 (−0.0591, +0.0462)	0.8046	−0.013	−0.0023 (−0.0480, +0.0433)	0.9174
DA ratio max (2 mm)	+0.056	+0.0261 (−0.0570, +0.1092)	0.5302	+0.021	+0.0293 (−0.3386, +0.3973)	0.8721	−0.050	−0.0649 (−0.4067, +0.2770)	0.7019
Radius (mm)	−0.358*	−0.3568 (−0.6238, −0.0899)	0.0099	−0.098	−0.3797 (−1.5576, +0.7982)	0.5161	−0.064	−0.2351 (−1.0589, +0.5886)	0.5654
Integrated radius (mm^-1^)	+0.335*	+0.4407 (+0.1731, +0.7083)	0.0018	+0.104	+0.4282 (−0.4739, +1.3303)	0.3409	+0.071	+0.2299 (−0.4082, +0.8679)	0.4688
Max inverse radius (mm^-1^)	+0.439*	+0.0108 (+0.0046, +0.0171)	0.001	−0.082	−0.0084 (−0.0452, +0.0285)	0.6465	+0.117	+0.0070 (−0.0103, +0.0244)	0.4168
SP-A1 (mmHg/mm)	+0.055	+1.1412 (−1.7938, +4.0762)	0.4376	−0.158*	−11.0391 (−21.8001, −0.2781)	0.0447	+0.080	+4.7966 (−8.4124, +18.0056)	0.4653
SSI	−0.284	−0.0485 (−0.0971, +0.0002)	0.0509	−0.140	−0.0827 (−0.2828, +0.1174)	0.4061	−0.105	−0.0516 (−0.2138, +0.1106)	0.522

* indicate P < 0.05. WTR, with-the-rule astigmatism; ATR, against-the-rule astigmatism; Oblique = oblique astigmatism.

## 4 Discussion

This study clarifies the relationships between astigmatism type and *in vivo* biomechanical parameters after adjustment for confounding factors. The results showed a significant difference in SP-A1 among astigmatism types (P = 0.0071), with higher values in the WTR group than in the ATR and Oblique groups. Analysis of astigmatic vector components further showed that a greater WTR component was associated with higher SP-A1, Integrated Radius, and HC dArc length, and lower Radius. These findings extend previous observations linking corneal curvature with Corvis ST parameters and indicate that astigmatism type may affect *in vivo* corneal biomechanical measurements, providing new evidence for the precise interpretation of biomechanical reports and accurate evaluation of *in vivo* biomechanical properties in patients.

Notably, SP-A1 was the only parameter that differed among the four groups, with higher values in the WTR group than in the ATR and Oblique groups. SP-A1 is a stiffness index at first applanation introduced by Roberts et al. (2017). It is calculated as $SP-A1=\frac{adjAP1-bIOP}{A1\;DeflAmp\;L}$ , where adjAP1 represents the actual pressure on the cornea generated by Corvis ST during first applanation, corrected for air pulse decay, bIOP represents biomechanically corrected IOP, and A1 DeflAmp L represents the corneal deflection amplitude at first applanation (Aoki et al., 2023). Corvis ST records dynamic corneal deformation along the central approximately 8 mm horizontal meridian using a high-speed Scheimpflug camera (Wang et al., 2021). In WTR eyes, the vertical meridian is steeper, and the horizontal meridian is flatter. Therefore, when the numerator (adjAP1 – bIOP) is similar after adjusting for confounders, the horizontal measurement section in WTR eyes corresponds to the flatter meridian, has a shorter apex displacement to reach first applanation (a smaller denominator for A1 DeflAmp L), resulting in higher SP-A1. These findings suggest that although SP-A1 is a reliable biomechanical parameter for preoperative refractive evaluation (Huo et al., 2026; Ren et al., 2021; Huo et al., 2023; Miao et al., 2024), in cases of high WTR eyes, it may be overestimated due to measurement error, which could partially mask the risk of early keratoconus or other ectatic diseases. However, this hypothesis requires further validation in populations with corneal ectasia and suspected keratoconus.

In the correlation analyses, multivariable linear regression controlling for J45, age, CCT, and IOP showed that J0 was independently and positively correlated with Integrated Radius, HC dArc length, and SP-A1 and independently and negatively correlated with Radius and SSI. The increase in Integrated Radius and HC dArc length, and decrease in Radius were directionally consistent, and all three parameters were derived from analysis of the horizontal section deformation profile during the air pulse process. Radius reflects the radius of curvature at the corneal apex at maximum concavity, Integrated Radius reflects the calculus of the radius of the reverse concavity, and HC dArc length reflects the Arc length change from the initial state to the highest concavity (Jędzierowska and Koprowski, 2019). An increase in J0 indicates a stronger WTR component, with the horizontal scanning section closer to the relatively flatter meridian. Therefore, a larger peripheral region may participate in deformation to reach the highest concavity, resulting in a larger Integrated Radius area and a greater change in HC dArc length. SSI is a material-related parameter calculated by finite element models (Eliasy et al., 2019). The negative correlation between J0 and SSI may be explained by the fact that SSI calculation depends on DCR parameters. Since differences in astigmatism axis can affect the dynamic corneal response, this may indirectly influence the SSI value. In multivariable linear regression, J45 was independently and negatively correlated with A1 time and A2 velocity. J45 represents the oblique astigmatic component. With increasing J45, the principal meridian approaches the oblique axis and the horizontal direction becomes relatively flatter, which may shorten the time to first applanation and affect rebound velocity. This relationship should be investigated further. Therefore, in eyes with high WTR astigmatism, it should be recognized that parameters such as SP-A1, Integrated Radius, HC dArc length, and Radius may be affected. Greater attention could be paid to parameters less influenced by astigmatic characteristics to reduce measurement-related interference in clinical decisions, such as early ectasia interpretation and refractive surgery risk evaluation.

The subgroup analyses were consistent with the vector component results. In the WTR group, cylinder power was positively correlated with Integrated Radius, Max inverse radius, and HC dArc length and negatively correlated with Radius, suggesting that higher WTR cylinder power was correlated with more pronounced deformation at highest concavity. In the ATR group, each 1D increase in cylinder power was associated with an estimated 11.04 mmHg/mm decrease in SP-A1, which was consistent with the steeper horizontal meridian in ATR eyes. This remind that, when interpreting SP-A1 in eyes with high magnitude astigmatism, it is important to distinguish a genuine reduction in corneal stiffness from the measurement-related decrease caused by corneal geometry. No significant correlation was observed in the Oblique group, possibly because the horizontal component of the oblique principal meridian was small and did not show an evident difference. Previous studies by Asaoka et al. and Salouti et al. found that corneal curvature correlated with multiple Corvis ST parameters (Asaoka et al., 2015; Salouti et al., 2020). Li et al. found differences in A1 time, Radius, SP-A1, Integrated Radius, and other parameters among astigmatism types in patients with cataract (Li et al., 2025). Chu et al. found that central corneal radius of curvature was independently correlated with SSI (Chu et al., 2023). These studies further support the reliability of the present findings and collectively indicate that Corvis ST parameters do not simply reflect corneal material and mechanical properties. Their outputs are also affected by corneal astigmatism magnitude.

This study had several limitations. First, ATR and Oblique astigmatism are less common than WTR astigmatism in clinical practice. A previous study of myopic population in China reported that WTR astigmatism accounted for nearly 90% of astigmatism types (Yang et al., 2022). This distribution resulted in relatively small subgroup sample sizes and a limited range of cylinder power in the present study, which may have obscured the effects of different axes and magnitudes on DCR parameters. Residual confounding by cylinder magnitude possibility also cannot be excluded. Future analyses should include larger multicenter samples. Second, this study included only relatively healthy young patients undergoing preoperative screening for refractive surgery. Corneal biomechanics change with age (Guo et al., 2024; Tahsini et al., 2025), and astigmatism type may shift from WTR toward ATR with advancing age (Beesley and Elliott, 2024). The findings therefore should not be directly generalized to other age groups or populations with ocular or systemic diseases. Future studies should include participants across different age groups and with different ocular and systemic diseases to broaden the applicability of the conclusions.

## 5 Conclusion

*In vivo* biomechanical measurements are affected by different types of astigmatism. SP-A1 was a sensitive parameter across astigmatism types and was higher in WTR than in ATR or Oblique eyes. The observed pattern may be largely driven by SP-A1 and selected vector-related associations rather than by widespread changes across the biomechanical profile. Therefore, the influence of astigmatism should be considered in clinical interpretation. Exploratory vector analysis further showed that as J0 increased (the principal astigmatic meridian became more WTR oriented and cylinder power increased), SP-A1, Integrated Radius, and HC dArc length increased, whereas Radius decreased. This study suggests that astigmatism type and cylinder power could be considered when analyzing *in vivo* biomechanical measurement results, providing a theoretical basis for more precise clinical interpretation of corneal biomechanics.

## Data Availability

The dataset used and analyzed in this study is available from the corresponding author upon reasonable request.
