# Supplementary material for: Analysis of astigmatism type influence on in vivo corneal biomechanical measurements
Source: Front Bioeng Biotechnol. 2026 Sep 1;14:1903049. doi: 10.3389/fbioe.2026.1903049 (PMC13574804; doi:10.3389/fbioe.2026.1903049)
Supplement: Supplementary file 1 [file Table1.docx]

**Supplementary Table 1.** Definitions of the 15 DCR parameters.

| Parameter | Definition |
| --- | --- |
| A1 and A2 time (ms) | Time to reach the first and second applanation |
| HC time (ms) | Time to reach the highest concavity |
| A1 and A2 velocity (m/s) | Velocity at the first and second applanation |
| A1 and A2 dArc length (mm) | Arc length change from the initial state to the first and second applanation |
| HC dArc length (mm) | Arc length change from the initial state to the highest concavity |
| DA ratio max (1 mm / 2 mm) | Corneal deformation ratio between the corneal apex and corneal apex within 1 mm / 2 mm |
| Radius (mm) | Radius of the curvature at the corneal apex at maximum concavity |
| Integrated radius (mm⁻¹) | Calculus of the radius of the reverse concavity |
| Max inverse radius (mm⁻¹) | Maximum value of the radius of the reverse concave surface |
| SP-A1 (mmHg/mm) | Stiffness parameter at first applanation |
| SSI (dimensionless) | Stress-strain index estimating corneal material stiffness with reduced dependence on IOP and CCT |

DCR = dynamic corneal response; IOP = intraocular pressure; CCT = central corneal thickness.

**Supplementary Table 2.** Correlations of demographic and ocular biometric variables with Corvis ST dynamic corneal response parameters.

**A. Age, CCT, IOP, and SER.**

| DCR parameter | Age (n = 155) | CCT (n = 155) | IOP (n = 155) | SER (n = 155) |
| --- | --- | --- | --- | --- |
| A1 time (ms) | rho=+0.076 (1.0000) | rho=+0.338^*^ (0.0013) | rho=+0.988^*^ (<0.0001) | rho=-0.068 (1.0000) |
| A2 time (ms) | rho=-0.054 (1.0000) | rho=-0.181 (0.5916) | rho=-0.866^*^ (<0.0001) | rho=+0.053 (1.0000) |
| HC time (ms) | rho=+0.080 (1.0000) | rho=+0.027 (1.0000) | rho=+0.003 (1.0000) | rho=-0.072 (1.0000) |
| A1 velocity (m/s) | rho=+0.073 (1.0000) | rho=-0.301^*^ (0.0076) | rho=-0.893^*^ (<0.0001) | rho=+0.012 (1.0000) |
| A2 velocity (m/s) | rho=+0.027 (1.0000) | r=+0.115 (1.0000) | rho=+0.651^*^ (<0.0001) | r=+0.084 (1.0000) |
| A1 dArc length (mm) | rho=-0.249^*^ (0.0264) | rho=-0.266^*^ (0.0336) | rho=-0.429^*^ (<0.0001) | rho=+0.230 (0.0589) |
| A2 dArc length (mm) | rho=-0.140 (1.0000) | rho=-0.405^*^ (<0.0001) | rho=-0.111 (1.0000) | rho=+0.083 (1.0000) |
| HC dArc length (mm) | rho=-0.064 (1.0000) | r=-0.183 (0.5555) | rho=+0.397^*^ (<0.0001) | r=+0.090 (1.0000) |
| DA ratio max (1 mm) | rho=-0.173 (0.4724) | r=-0.523^*^ (<0.0001) | rho=-0.601^*^ (<0.0001) | r=+0.021 (1.0000) |
| DA ratio max (2 mm) | rho=-0.067 (1.0000) | r=-0.541^*^ (<0.0001) | rho=-0.680^*^ (<0.0001) | r=+0.043 (1.0000) |
| Radius (mm) | rho=+0.129 (1.0000) | rho=+0.372^*^ (0.0002) | rho=+0.378^*^ (<0.0001) | rho=+0.043 (1.0000) |
| Integrated radius (mm⁻¹) | rho=-0.092 (1.0000) | r=-0.489^*^ (<0.0001) | rho=-0.708^*^ (<0.0001) | r=+0.028 (1.0000) |
| Max inverse radius (mm⁻¹) | rho=+0.005 (1.0000) | rho=-0.386^*^ (<0.0001) | rho=-0.218 (0.0968) | rho=-0.003 (1.0000) |
| SP-A1 (mmHg/mm) | rho=+0.105 (1.0000) | r=+0.655^*^ (<0.0001) | rho=+0.794^*^ (<0.0001) | r=-0.039 (1.0000) |
| SSI | rho=+0.194 (0.2343) | r=+0.094 (1.0000) | rho=+0.378^*^ (<0.0001) | r=+0.144 (1.0000) |

**Supplementary Table 2 (continued).**

**B. K1, K2, AL, sex, and myopia severity.**

| DCR parameter | K1 (n = 155) | K2 (n = 155) | AL (n = 155) | Sex (n = 155) | Myopia severity (n = 155) |
| --- | --- | --- | --- | --- | --- |
| A1 time (ms) | rho=+0.175 (0.7509) | rho=+0.163 (0.9948) | rho=-0.018 (1.0000) | r_rb_=-0.036 (1.0000) | rho=+0.061 (1.0000) |
| A2 time (ms) | rho=-0.045 (1.0000) | rho=-0.048 (1.0000) | r=-0.146 (1.0000) | r_rb_=+0.198 (0.5443) | rho=-0.043 (1.0000) |
| HC time (ms) | rho=+0.034 (1.0000) | rho=+0.040 (1.0000) | rho=-0.105 (1.0000) | r_rb_=+0.118 (0.0717) | rho=+0.024 (1.0000) |
| A1 velocity (m/s) | rho=-0.061 (1.0000) | rho=-0.072 (1.0000) | rho=-0.001 (1.0000) | r_rb_=+0.236 (0.1891) | rho=-0.001 (1.0000) |
| A2 velocity (m/s) | r=-0.030 (1.0000) | r=-0.029 (1.0000) | r=-0.077 (1.0000) | r_pb_=-0.200 (0.2034) | rho=-0.127 (1.0000) |
| A1 dArc length (mm) | rho=-0.135 (0.0950) | rho=-0.231 (0.1301) | rho=+0.082 (1.0000) | r_rb_=-0.243 (0.1433) | rho=-0.214 (0.1122) |
| A2 dArc length (mm) | rho=-0.186 (0.5560) | rho=-0.174 (0.7489) | rho=+0.100 (1.0000) | r_rb_=-0.112 (1.0000) | rho=-0.113 (1.0000) |
| HC dArc length (mm) | r=-0.163 (1.0000) | r=-0.113 (1.0000) | r=+0.054 (1.0000) | r_pb_=-0.100 (1.0000) | rho=-0.108 (1.0000) |
| DA ratio max (1 mm) | r=+0.217 (0.0522) | r=+0.217 (0.2106) | r=-0.230 (0.3517) | r_pb_=+0.064 (1.0000) | rho=-0.020 (1.0000) |
| DA ratio max (2 mm) | r=+0.208 (0.0669) | r=+0.206 (0.2922) | r=-0.250 (0.2008) | r_pb_=+0.106 (1.0000) | rho=-0.022 (1.0000) |
| Radius (mm) | rho=-0.103 (1.0000) | rho=-0.141 (1.0000) | rho=-0.081 (1.0000) | r_rb_=-0.113 (1.0000) | rho=-0.041 (1.0000) |
| Integrated radius (mm⁻¹) | r=+0.043 (1.0000) | r=+0.131 (1.0000) | r=-0.082 (1.0000) | r_pb_=+0.112 (1.0000) | rho=-0.015 (1.0000) |
| Max inverse radius (mm⁻¹) | rho=+0.152 (1.0000) | rho=+0.217 (0.2075) | rho=-0.073 (1.0000) | r_rb_=+0.123 (1.0000) | rho=+0.054 (1.0000) |
| SP-A1 (mmHg/mm) | r=+0.018 (1.0000) | r=+0.087 (1.0000) | r=+0.123 (1.0000) | r_rb_=-0.095 (1.0000) | rho=+0.036 (1.0000) |
| SSI | r=-0.078 (1.0000) | r=-0.224 (0.1676) | r=-0.230 (0.3558) | r_rb_=-0.041 (1.0000) | rho=-0.176 (0.4208) |

Values are Pearson (r), Spearman (rho), point-biserial (rpb), or rank-biserial (rrb) correlation coefficients; Bonferroni-adjusted P values are shown in parentheses. * indicates an adjusted P value < 0.05. AL = axial length; CCT = central corneal thickness; IOP = intraocular pressure; K1/K2 = flat/steep keratometry; SER = spherical equivalent refraction.

**Supplementary Table 3.** SP-A1 comparisons across each astigmatism type.

| Comparison | Mean diff (mmHg/mm) | 95% CI | p (LSD) |
| --- | --- | --- | --- |
| WTR vs No astigmatism | +2.29 | (-1.34, +5.91) | 0.215 |
| ATR vs No astigmatism | -2.60 | (-6.43, +1.22) | 0.181 |
| Oblique vs No astigmatism | -3.04 | (-6.83, +0.75) | 0.115 |
| WTR vs ATR | +4.89 | (+1.46, +8.33) | 0.0057* |
| WTR vs Oblique | +5.32 | (+1.95, +8.70) | 0.0020* |
| ATR vs Oblique | -0.43 | (-4.07, +3.20) | 0.815 |

* indicate P < 0.05. WTR = with-the-rule astigmatism; ATR = against-the-rule astigmatism; Oblique = oblique astigmatism; LSD = Fisher's least significant difference method.

**Supplementary Table 4.** Multivariable linear regression of Thibos J0/J45 vector components on dynamic corneal response parameters.

| Parameter | Standardized β (J0) | B (95% CI) (J0) | p value (J0) | Standardized β (J45) | B (95% CI) (J45) | p value (J45) |
| --- | --- | --- | --- | --- | --- | --- |
| A1 time (ms) | +0.008 | +0.0069 (-0.0145, +0.0283) | 0.5257 | -0.033* | -0.0444 (-0.0785, -0.0103) | 0.011 |
| A2 time (ms) | -0.039 | -0.0482 (-0.1803, +0.0840) | 0.4725 | -0.028 | -0.0549 (-0.2654, +0.1556) | 0.6069 |
| HC time (ms) | -0.009 | -0.0109 (-0.2338, +0.2119) | 0.9228 | -0.059 | -0.1153 (-0.4702, +0.2395) | 0.5217 |
| A1 velocity (m/s) | -0.060 | -0.0036 (-0.0083, +0.0012) | 0.1399 | -0.042 | -0.0040 (-0.0116, +0.0036) | 0.2994 |
| A2 velocity (m/s) | -0.045 | -0.0042 (-0.0169, +0.0085) | 0.5191 | -0.156* | -0.0233 (-0.0435, -0.0031) | 0.0242 |
| A1 dArc length (mm) | +0.106 | +0.0007 (-0.0003, +0.0018) | 0.1719 | -0.046 | -0.0005 (-0.0022, +0.0012) | 0.5512 |
| A2 dArc length (mm) | +0.028 | +0.0005 (-0.0023, +0.0032) | 0.7483 | +0.094 | +0.0024 (-0.0020, +0.0068) | 0.2805 |
| HC dArc length (mm) | +0.223* | +0.0138 (+0.0042, +0.0234) | 0.0051 | +0.045 | +0.0045 (-0.0108, +0.0197) | 0.5634 |
| DA Ratio Max (1 mm) | -0.043 | -0.0052 (-0.0213, +0.0109) | 0.5234 | -0.018 | -0.0034 (-0.0290, +0.0222) | 0.7943 |
| DA Ratio Max (2 mm) | -0.064 | -0.0601 (-0.1784, +0.0581) | 0.3167 | -0.032 | -0.0481 (-0.2365, +0.1403) | 0.6145 |
| Radius (mm) | -0.182* | -0.4350 (-0.8104, -0.0597) | 0.0234 | -0.103 | -0.3927 (-0.9905, +0.2051) | 0.1963 |
| Integrated Radius (mm⁻¹) | +0.196* | +0.5170 (+0.2080, +0.8260) | 0.0012 | +0.013 | +0.0528 (-0.4394, +0.5449) | 0.8325 |
| Max inverse radius (mm⁻¹) | +0.167 | +0.0090 (-0.0001, +0.0182) | 0.053 | -0.063 | -0.0054 (-0.0200, +0.0091) | 0.4628 |
| SP-A1 (mmHg/mm) | +0.101* | +4.4345 (+0.4070, +8.4619) | 0.0312 | -0.067 | -4.6931 (-11.1077, +1.7214) | 0.1504 |
| SSI | -0.181* | -0.0691 (-0.1332, -0.0049) | 0.035 | -0.023 | -0.0138 (-0.1160, +0.0884) | 0.7902 |

* indicate P < 0.05.

**Supplementary Table 5.** Diagnostic assessment of the multivariable linear regression models

**Panel A. Multicollinearity diagnostics**

| Predictor | VIF |
| --- | --- |
| J0 | 1.268 |
| J45 | 1.263 |
| age | 1.128 |
| CCT | 1.137 |
| IOP | 1.232 |

**Panel B. Residual, homoscedasticity, and influence diagnostics**

| Outcome | Shapiro– Wilk W | P value | Breusch– Pagan χ² | P value | Maximum Cook’s D | Cook’s D >1, n |
| --- | --- | --- | --- | --- | --- | --- |
| A1 time (ms) | 0.980 | 0.021 | 6.852 | 0.232 | 0.129 | 0 |
| A2 time (ms) | 0.818 | <0.001 | 2.887 | 0.717 | 0.214 | 0 |
| HC time (ms) | 0.968 | 0.001 | 6.714 | 0.243 | 0.151 | 0 |
| A1 velocity (m/s) | 0.989 | 0.281 | 11.024 | 0.051 | 0.137 | 0 |
| A2 velocity (m/s) | 0.986 | 0.136 | 4.193 | 0.522 | 0.075 | 0 |
| A1 dArc length (mm) | 0.964 | <0.001 | 7.729 | 0.172 | 0.055 | 0 |
| A2 dArc length (mm) | 0.836 | <0.001 | 3.676 | 0.597 | 0.155 | 0 |
| HC dArc length (mm) | 0.982 | 0.039 | 7.508 | 0.186 | 0.094 | 0 |
| DA Ratio Max (1 mm) | 0.981 | 0.029 | 5.004 | 0.415 | 0.096 | 0 |
| DA Ratio Max (2 mm) | 0.988 | 0.201 | 3.361 | 0.644 | 0.088 | 0 |
| Radius (mm) | 0.979 | 0.019 | 5.874 | 0.319 | 0.087 | 0 |
| Integrated Radius (mm⁻¹) | 0.970 | 0.002 | 4.898 | 0.428 | 0.202 | 0 |
| Max inverse radius (mm⁻¹) | 0.896 | <0.001 | 5.350 | 0.375 | 0.142 | 0 |
| SP-A1 (mmHg/mm) | 0.951 | <0.001 | 2.964 | 0.706 | 0.155 | 0 |
| SSI | 0.990 | 0.358 | 3.789 | 0.580 | 0.098 | 0 |

A Cook’s distance >1 was used to flag a potentially influential observation.
